# Systemic strategies to prevent early diabetic retinopathy: targeting polyunsaturated fatty acid metabolism and eicosanoid signaling

**DOI:** 10.3389/fmed.2026.1802443

**Published:** 2026-04-13

**Authors:** Mohammad S. Aqil, Marcus Yaldo, Gieth Alahdab, Charlene Hsiung, Amany Tawfik, Mohamed Al-Shabrawey

**Affiliations:** 1 Eye Research Center and Foundational Medical Studies, Oakland University William Beaumont School of Medicine, Rochester, MI, United States; 2 Eye Research Institute, Oakland University, Rochester, MI, United States

**Keywords:** 12/15-lipoxygenase, bioactive lipids, diabetic retinopathy, dietary (food) supplements, eicosanoids, homocysteine, polyunsaturated fatty acids (PUFA)

## Abstract

Diabetic retinopathy (DR) is a leading cause of preventable vision loss, yet current therapies primarily address late, VEGF-driven vascular complications rather than early upstream drivers. Emerging evidence indicates that early DR originates from metabolic stress within the retinal neurovascular unit, where dysregulated lipid metabolism, oxidative stress, and inflammation precede visible microvascular damage. Disturbances in polyunsaturated fatty acid (PUFA) metabolism, together with related metabolic stressors such as elevated homocysteine (Hcy), drive lipid dysregulation, oxidative stress, and inflammation preceding visible microvascular damage, promoting endothelial dysfunction and blood–retinal barrier (BRB) breakdown. Hyperglycemia shifts retinal lipid composition toward oxidation-prone omega-6 PUFAs and activates lipoxygenase (LOX), cyclooxygenase (COX), and cytochrome P450 (CYP450) eicosanoid pathways. LOX-derived metabolites such as 12- and 15-HETE stimulate NADPH oxidase, disrupt tight junctions, and promote inflammatory signaling in endothelial and Müller cells. COX-2–driven prostaglandin E2 signaling increases vascular permeability, while CYP450 metabolites and their soluble epoxide hydrolase (sEH) derived products exert context-dependent effects on vascular integrity. Elevated Hcy further enhances oxidative stress and NF-κB activation, amplifying PUFA-mediated inflammatory signaling. These mechanisms identify modifiable upstream targets that complement glycemic control. Higher dietary omega-3 intake and lower omega-6:omega-3 ratios are associated with reduced DR risk, particularly in well-controlled diabetes. Omega-3–rich diets, exercise, and correction of folate and B-vitamin deficiencies may help improve systemic inflammation and retinal barrier integrity. Integrating lipid pathway modulation, nutritional support, and metabolic control with careful ocular monitoring may help slow the progression of DR before irreversible blindness occurs.

## Introduction

Diabetic retinopathy (DR) is a leading cause of preventable vision loss worldwide. About one-third of people with diabetes show DR, and one-third of those have vision-threatening disease such as proliferative DR or diabetic macular edema (DME) (1). Current treatments primarily target downstream vascular endothelial growth factor (VEGF)-driven vascular complications and inflammation.

Despite the growing role of anti-VEGF therapy across the spectrum of DR, its preventive use remains complex. In a 4-years randomized trial involving eyes with moderate to severe non-proliferative diabetic retinopathy (NPDR) without center-involved diabetic macular edema (CI-DME), prophylactic intravitreal aflibercept (anti-VEGF) significantly reduced progression to proliferative diabetic retinopathy (PDR) or CI-DME (2). This finding highlights the capacity of anti-VEGF therapy to modify the anatomic course of disease even before vision-threatening complications develop. However, this reduction in structural progression did not translate into an improvement in mean visual acuity over the study period, underscoring both the expanding therapeutic relevance and the limitations of anti-VEGF therapy across different stages of DR. While anti-VEGF agents remain central to the management of CI-DME and are increasingly used in PDR to control neovascularization, the evidence does not support routine prophylactic treatment for all NPDR eyes. Instead, the data suggest that early anti-VEGF intervention may be most appropriate for selected high-risk NPDR patients, where the goal is to reduce progression to more advanced disease rather than to achieve immediate visual acuity gains (2).

Emerging biology highlights the importance of identifying upstream targets that contribute to the pathogenesis of DR. Beyond hyperglycemia, dysregulated lipid metabolism, oxidative stress, and inflammation drive early disease (3–5). For example, diabetes remodels retinal polyunsaturated fatty acids (PUFA) substrate pools and increases both enzymatic and non-enzymatic PUFA oxidation and generation of eicosanoids (bioactive lipids) (6–9) that could play a role in the pathogenesis of DR. Specific pathway outputs and representative mediators are detailed in the section “Retinal Eicosanoid Pathways.” In addition to PUFA, the role of elevated homocysteine (Hcy) or hyperhomocysteinemia (HHcy) has been documented in human DR and experimental diabetes through enhancing oxidative and endoplasmic reticulum stress and leukostasis (10). Interestingly, evidence also suggests that HHcy dysregulates PUFA-derived eicosanoid levels (11). An excess of omega-6 relative to omega-3 PUFA can promote pro-inflammatory eicosanoids and vascular injury (12–14). Together, these findings position lipid mediator biology, Hcy metabolism, and oxidative signaling as potential upstream targets that complement glycemic control and may help slow disease progression before irreversible vision loss occurs.

This review focuses on PUFA metabolism and eicosanoid signaling as upstream drivers of early DR, and highlights selected modifiable metabolic factors, including Hcy, that may enhance lipid-mediated oxidative and inflammatory signaling within the retinal neurovascular unit.

## Pathogenesis of early diabetic retinopathy

Early DR represents a disorder of the neurovascular unit in which Müller cells, pericytes, endothelial cells, and neurons exhibit coordinated dysfunction before overt clinical lesions. Hyperglycemia induces metabolic and cellular stress that drives VEGF signaling, mitochondrial superoxide production, and induces persistent epigenetic changes, thereby priming inflammatory signaling in glia and endothelial cells (15). Müller cell gliosis increases local cytokine release and weakens the inner BRB (16). In parallel, early pericyte dropout similarly destabilizes retinal capillaries and is widely considered a prerequisite for microaneurysm formation (17). Simultaneously, endothelial cells adopt a pro-inflammatory, pro-adhesive phenotype that contributes to leukostasis with reduced nitric oxide bioavailability and sluggish capillary flow (7, 18, 19). At the tissue level, oxidative stress, inflammation, and vascular instability converge to drive early disease progression.

In human retinal endothelial cells, high glucose is associated with lipidomic shifts consistent with increased PUFA oxidation that promote NADPH oxidase activation and tight-junction disruption; conversely, genetic or pharmacologic suppression of lipid-oxidation pathways attenuates permeability and inflammatory signaling in diabetic models (6, 8). Neuronal stress and retinal ganglion cell dysfunction arise early in disease pathogenesis and can precede structural vascular lesions, aligning with functional deficits in diet-induced models (20). In a high-fat diet mouse model, retinal stress-kinase activation and inflammasome signaling appeared by 3 months. By 6 months, electroretinography showed delayed responses and reduced amplitudes proportional to glucose intolerance, and by 12 months, classic microvascular lesions with vascular leakage had developed (20). Importantly, these findings indicate that neuroinflammatory and functional deficits precede overt vascular lesions, reframing early DR as more than a purely microvascular disorder (20). This feed-forward loop of oxidative injury, leukostasis, and barrier failure links systemic metabolic tone to local ischemia and neurodegeneration, motivating upstream prevention strategies. Furthermore, human omics data align with this model (21, 22). Stage-specific retinal RNA-seq has revealed alterations in Hippo and gap-junction signaling, modulation of sphingolipid and cGMP–PKG pathways, and single-cell evidence of retinal ganglion cell loss alongside Müller glial metabolic reprogramming, highlighting neuroglial and lipid-metabolic contributions beyond classical vasculopathy (23).

Chronic hyperglycemia and dyslipidemia reshape vascular communication in the retina by activating endothelial and glial cells to express adhesion molecules and cytokines that recruit circulating leukocytes. In early disease, microglia adopt a pro-inflammatory phenotype, releasing interleukin-1β, tumor necrosis factor alpha (TNF-α), and other mediators that amplify endothelial activation, compromising the inner BRB (24). These adherent leukocytes impede capillary flow and contribute further to BRB destabilization (22). As nitric oxide bioavailability falls and oxidative and LOX-linked signaling intensifies, microvascular permeability becomes patchy and uneven, consistent with early lesions that may later manifest clinically as microaneurysms and hemorrhages (22). Together, these leukocyte–endothelium interactions bridge systemic inflammation with local ischemia, illustrating how early correction of metabolic and lipid mediator imbalances may reduce structural risk long before intravitreal therapy becomes necessary (20, 25).

### Hyperglycemia-linked pathways

Chronic hyperglycemia increases flux through the polyol pathway, where aldose reductase reduces glucose to sorbitol using NADPH (26). Sorbitol can accumulate intracellularly and contribute to osmotic stress, while NADPH depletion impairs glutathione regeneration and antioxidant defenses (27), thereby amplifying oxidative stress. Downstream conversion of sorbitol to fructose further perturbs cellular redox balance, reinforcing endothelial dysfunction and barrier vulnerability in concert with PKC activation, AGE-RAGE signaling, and hexosamine pathway flux (15, 28). These mechanisms converge on mitochondrial superoxide generation and robust epigenetic changes that underlie metabolic memory and sustain inflammatory signaling even after later glycemic improvement (15). LOX-linked lipid changes intersect with these pathways by amplifying NADPH oxidase activity and destabilizing junctional integrity under high-glucose conditions (6, 8). Together, these interconnected metabolic and lipid-driven processes establish a persistent pro-oxidant, pro-inflammatory environment that drives progressive microvascular dysfunction, even following normalization of glycemia.

### Lipid remodeling, peroxidation, and ceramides

Diabetes substantially alters the retinal lipid landscape, including loss and remodeling of long-chain PUFAs. As these lipids undergo peroxidation, reactive aldehydes such as 4-hydroxynonenal accumulate, further amplifying oxidative stress and inflammatory signaling across both vascular and neural compartments (29). Ceramide accumulation and a ferroptotic redox state further heighten cellular vulnerability, weakening both capillary and neuronal integrity (30–32). In experimental models, interventions that limit lipid peroxidation or ferroptosis preserve vascular structure and neuronal viability, suggesting that early control of triglyceride flux and PUFA composition may meaningfully alter disease trajectory (30–32). Multi-omics profiling now places disturbed lipid metabolism at the hub of complication-prone tissues, including the retina, supporting the rationale for coupling standard metabolic care with therapies that restore a protective, pro-resolving eicosanoid balance (33). In a cohort of 379 adults with diabetes, higher dietary PUFA intake correlated with lower retinopathy odds and severity, while higher saturated fat intake was associated with greater severity, particularly among individuals with HbA1c < 7 percent (34). These observations suggest that dietary effects may be contingent on the broader context of systemic glycemic control.

### PPAR signaling as a lipid-sensing brake on retinal inflammation

Peroxisome proliferator-activated receptors (PPARs) are lipid-sensing nuclear receptors that link fatty-acid and eicosanoid cues to transcriptional programs regulating oxidative stress, inflammation, and barrier integrity (35). In the retina, reduced PPARγ signaling has been observed in experimental diabetes and oxygen-induced retinopathy and appears to coincide with heightened NF-κB activity. Restoration of PPARγ activity has been reported following NADPH oxidase inhibition or genetic deletion of NOX2, suggesting that oxidative signaling can suppress this anti-inflammatory transcriptional axis and thereby worsen endothelial activation and vascular leakage (36). Together, these findings position PPAR pathways as a mechanistic bridge between dysregulated lipid mediator tone and downstream inflammatory gene expression in early DR.

### Homocysteine as a metabolic regulator of PUFA-derived eicosanoids in DR

Homocysteine is a sulfur-containing amino acid formed during the metabolism of methionine, an essential amino acid obtained from dietary protein. In normal physiology, Hcy is metabolized via two primary pathways: remethylation and transsulfuration. In remethylation, Hcy is converted back to methionine by methionine synthase, which requires folate and vitamin B12 as cofactors. In the transsulfuration pathway, Hcy is irreversibly converted to cystathionine by cystathionine β-synthase (CBS), a reaction dependent on vitamin B6, ultimately leading to the formation of cysteine (37, 38). Disruptions in these pathways, due to nutritional deficiencies, genetic variants (e.g., MTHFR polymorphisms), or oxidative stress, can lead to elevated Hcy or HHcy.

**FIGURE 1:**
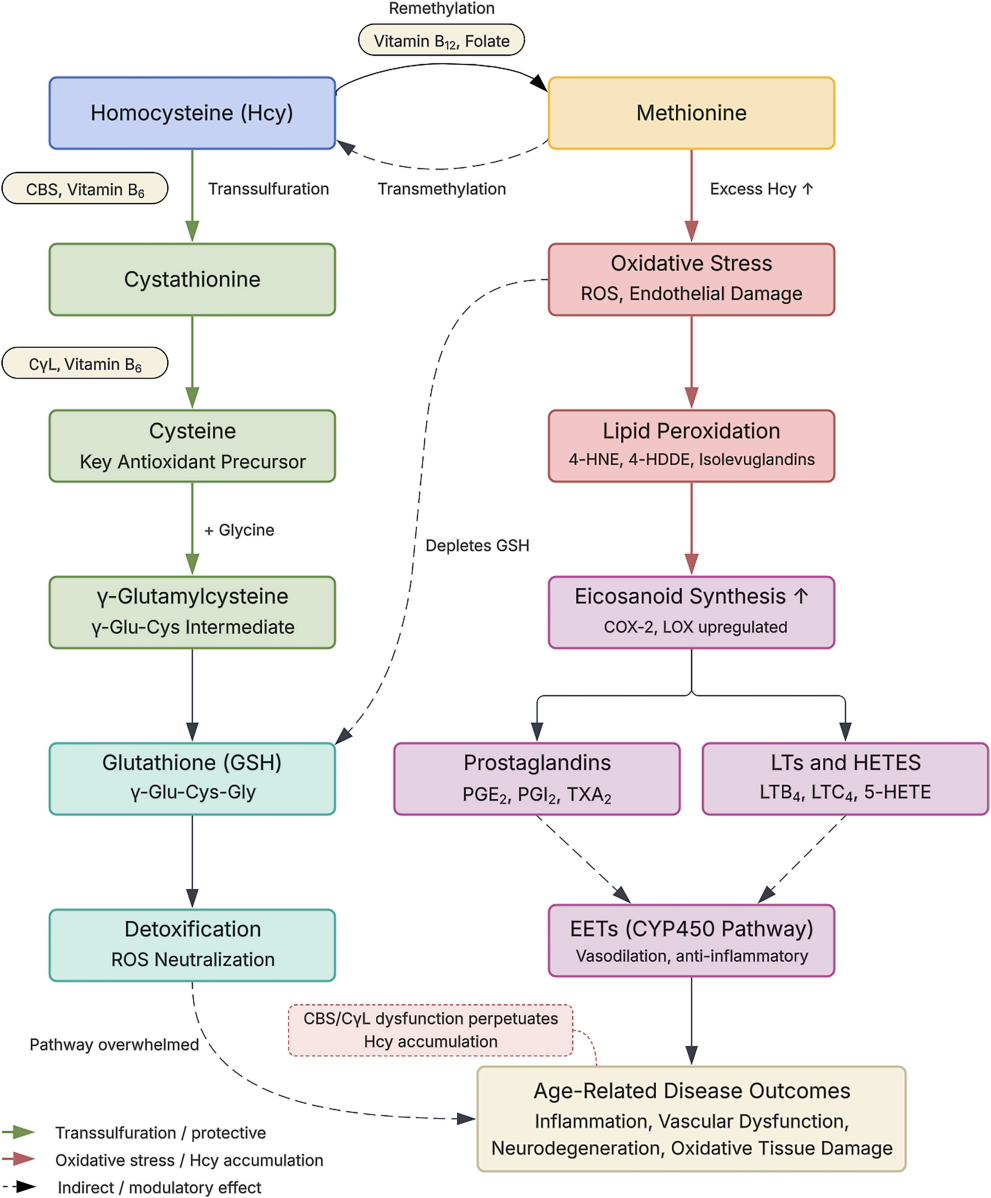
Link between homocysteine metabolism and bioactive lipid mediators. Homocysteine lies at the intersection of remethylation and transsulfuration pathways. Through remethylation, homocysteine is recycled to methionine, whereas through transsulfuration, it is converted to cystathionine and then cysteine, supporting γ-glutamylcysteine and glutathione (GSH) synthesis for antioxidant defense. When homocysteine accumulates, oxidative stress and lipid peroxidation increase, depleting GSH and promoting the formation of reactive lipid aldehydes and oxidized lipid derivatives. These changes are associated with upregulation of eicosanoid-generating pathways, increasing inflammatory lipid mediators including prostaglandins, leukotrienes, and HETEs, while altering CYP450-derived signaling. Collectively, disrupted one-carbon metabolism links homocysteine excess to inflammatory, oxidative, and vascular dysfunction.

Elevated Hcy has multiple vascular consequences relevant to DR. Mechanistically, HHcy activates NADPH oxidase, generating reactive oxygen species (ROS) that trigger NF-κB–mediated inflammatory signaling, upregulate COX and LOX enzymes (39, 40), and promote the production of pro-inflammatory eicosanoids such as PGE_2_, 12-HETE, and 15-HETE (12, 37, 38). HHcy also reduces endothelial nitric oxide bioavailability, impairs vasodilation, and disrupts the inner and outer blood-retinal barriers (37, 41), thereby increasing vascular permeability. Clinically, higher plasma Hcy levels correlate with both the presence and severity of diabetic retinopathy, with patients exhibiting elevated Hcy showing greater progression from non-proliferative to proliferative stages (42–44). Collectively, these data position Hcy metabolism as a modifiable upstream regulator of oxidative, inflammatory, and lipid-mediated signaling in the retina, highlighting its potential as a therapeutic target alongside glycemic control (Figure 1).

## Retinal eicosanoid pathways

As dietary lipids supply the substrates for eicosanoid biosynthesis, observational data linking higher PUFA and lower saturated fat intake to less DR in well-controlled diabetes align with a model in which precursor pools influence pro- and anti-inflammatory lipid mediators (34).

### COX pathway

Cyclooxygenase-2 (COX-2) is upregulated in the diabetic retina, increasing conversion of arachidonic acid into bioactive prostanoids that regulate vascular permeability and inflammation. Elevated prostaglandin E_2_ (PGE_2_) enhances VEGF expression, leukocyte adhesion, and barrier dysfunction. In streptozotocin-induced diabetic rats, intravitreal PGE_2_ or the EP_2_ receptor agonist butaprost accelerated vascular leakage and endothelial apoptosis, whereas EP_2_ antagonism attenuated these effects and suppressed cAMP/PKA/CREB and NLRP3 signaling, identifying EP_2_ as a key downstream mediator (45). Pharmacologic COX-2 inhibition reduces inflammatory signaling and vascular leakage in experimental models, supporting its role in early microvascular dysfunction (4, 25).

Beyond PGE_2_, COX enzymes generate four additional prostanoids: prostacyclin (PGI_2_), thromboxane A_2_ (TXA_2_), prostaglandin D_2_ (PGD_2_), and prostaglandin F_2_α (PGF_2_α); each exerts distinct vascular effects. PGI_2_ promotes vasodilation and inhibits platelet aggregation, whereas TXA_2_ enhances vasoconstriction and platelet activation. PGF_2_α has been implicated in angiogenic signaling, and PGD_2_ modulates immune responses (46, 47). Thus, the impact of COX activation in DR likely reflects the balance among prostanoid species and receptor signaling rather than isolated PGE_2_ elevation alone. Because prostanoids also support vascular homeostasis, broad COX inhibition poses translational challenges, highlighting the importance of selective or upstream lipid pathway modulation (11, 48).

### LOX pathway

In various experimental models of early DR, 12/15-LOX-derived eicosanoids such as 12-HETE and 15-HETE amplify leukocyte adhesion, vascular leakage, and pericyte loss. Elevated 12-LOX expressions and activity are evident in both human diabetic specimens and experimental diabetic mice, as well as in oxygen-induced retinopathy, while genetic or pharmacologic LOX inhibition reduces neovascularization and barrier breakdown (6–9). Bioactive lipid mediators, including LOX-derived eicosanoids, have been recognized as potent regulators of pathological retinal angiogenesis and inflammation, linking lipid signaling directly to neovascular remodeling (3). In retinal endothelial monolayers, exogenous 12/15-HETE activates NADPH oxidase and vascular endothelial growth factor receptor 2 (VEGFR-2) signaling, destabilizing intercellular junctions–an effect reversed by oxidase blockade (6). Under hyperglycemic conditions, a LOX-linked lipidomic profile drives reactive oxygen species generation, cytokine release, and tight-junction disruption that normalize with 12/15-LOX or oxidase inhibition (8). The high-affinity 12-HETE receptor GPR31, expressed in retinal cells, mediates endothelial inflammatory responses, positioning selective enzyme or receptor blockade as promising upstream strategies (49). Furthermore, the 12-HETE/GPR31 axis demonstrated enhanced inflammatory and oxidative responses in Müller glial cells (50). Protective counter-regulatory pathways also exist; pigment epithelium-derived factor mitigates 12/15-HETE–induced microvascular dysfunction and pericyte injury (8). Although no LOX-targeted ophthalmic therapy has reached clinical approval, these mechanistic insights define an emerging class of lipid-modulating interventions poised for translational exploration.

### CYP450 and soluble epoxide hydrolase (sEH)

**FIGURE 2:**
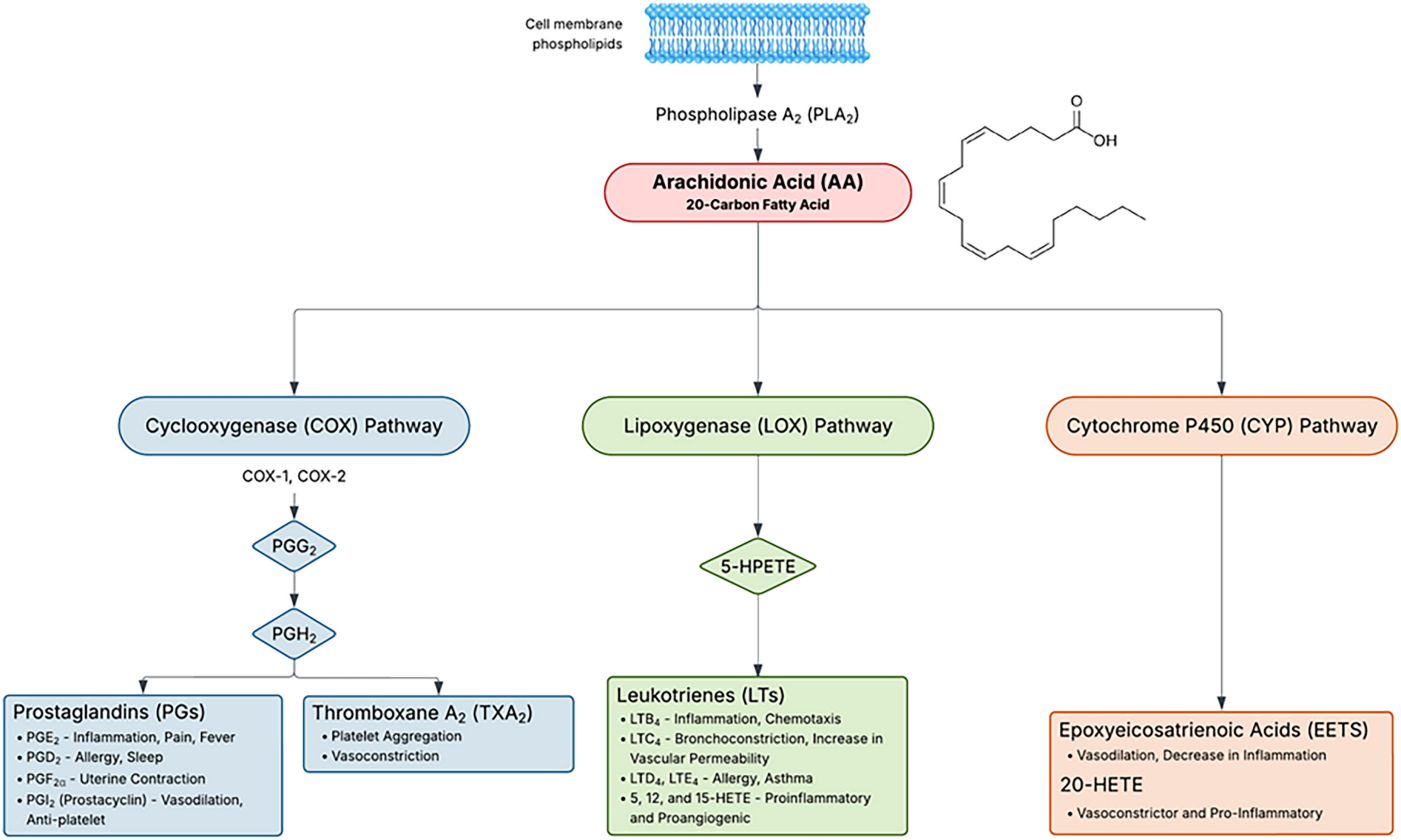
Arachidonic acid metabolism and eicosanoid pathways. Arachidonic acid (AA), released from membrane phospholipids by phospholipase A_2_ (PLA_2_), is metabolized through three major enzymatic pathways. Cyclooxygenase (COX-1/COX-2) generates prostaglandins (PGs) and thromboxane A_2_ (TXA_2_), which regulate inflammation, vascular tone, pain, fever, and platelet aggregation. Lipoxygenase (LOX) forms hydroperoxyeicosatetraenoic acids (HPETEs), leukotrienes (LTs), and HETEs, mediators involved in inflammation, chemotaxis, bronchoconstriction, and vascular permeability. Cytochrome P450 (CYP) produces epoxyeicosatrienoic acids (EETs) and 20-HETE, which exert context-dependent effects on vascular and inflammatory signaling. Together, these eicosanoid pathways coordinate inflammatory, vascular, and hemodynamic responses.

The cytochrome P450 lipid axis (Figure 2) contributes to DR, but the directionality of benefit can be context dependent. In the retina, CYP epoxygenases generate arachidonic-acid–derived epoxyeicosatrienoic acids (EETs) that are vasodilatory in systemic vessels yet, under diabetic and hypoxic retinal conditions, can potentiate VEGF signaling, angiogenesis, and vascular leak (51, 52). In parallel, CYP ω-hydroxylases produce 20-HETE, which is vasoconstrictive and pro-inflammatory, and inhibition of 20-HETE attenuates diabetes-induced decreases in retinal blood flow (53, 54). Soluble epoxide hydrolase (sEH) hydrolyzes EETs and epoxy-docosahexaenoic acid (DHA) species; for arachidonic acid, this yields less active dihydroxyeicosatrienoic acids (DiHETrEs), and for DHA it generates 19,20-dihydroxydocosapentaenoic acid (19,20-DHDP) (55, 56). In diabetes, retinal sEH and 19,20-DHDP increase, and 19,20-DHDP disrupts cholesterol-handling and cadherin-associated junctional complexes, weakening pericyte–endothelial adhesion and endothelial barrier integrity (56, 57). Consistent with this mechanism, multiple diabetic and vasoregression models report that pharmacologic sEH inhibition is protective, preserving epoxide bioavailability, limiting vascular leakage, and reducing pericyte dropout, whereas Müller-cell sEH overexpression can recapitulate retinopathy features (56, 58, 59). However, other data indicate that sEH suppression can be detrimental when renin–angiotensin signaling and EET biology intersect: Ang II increases retinal sEH via AT1 receptor signaling, AT1 blockade blunts this induction, exogenous 11,12-EET exacerbates Ang II–induced retinal albumin leakage, and sEH knockout potentiates diabetes-associated retinal leakage with increased VEGF and reduced tight junction proteins [zonula occludens-1 (ZO-1), occludin] (13). Collectively, these findings support a model in which the net effect of sEH modulation depends on cell compartment (endothelium vs. Müller glia), substrate balance (AA-derived EETs vs. DHA-derived epoxides and 19,20-DHDP), and concurrent renin–angiotensin system activation, which may explain why sEH blockade appears barrier-protective in some DR paradigms yet permissive to permeability signaling in others (13, 56, 58, 59).

### Specialized pro-resolving mediators (SPMs)

Specialized pro-resolving mediators (SPMs) derived from omega-3 PUFAs include E-series resolvins (RvE1), D-series resolvins (RvD1, RvD2), protectins (including neuroprotectin D1), maresins, and lipoxin A4 derived from arachidonic acid (60–62). In experimental retinal models, RvD1 reduces leukocyte adhesion and vascular leakage, while neuroprotectin D1 protects retinal pigment epithelial and neuronal cells from oxidative stress–induced apoptosis. Lipoxin A4 suppresses NF-κB activation and inflammatory cytokine expression, contributing to the resolution of retinal inflammation. These mediators actively restore immune balance rather than simply suppress inflammatory signaling.

**FIGURE 3:**
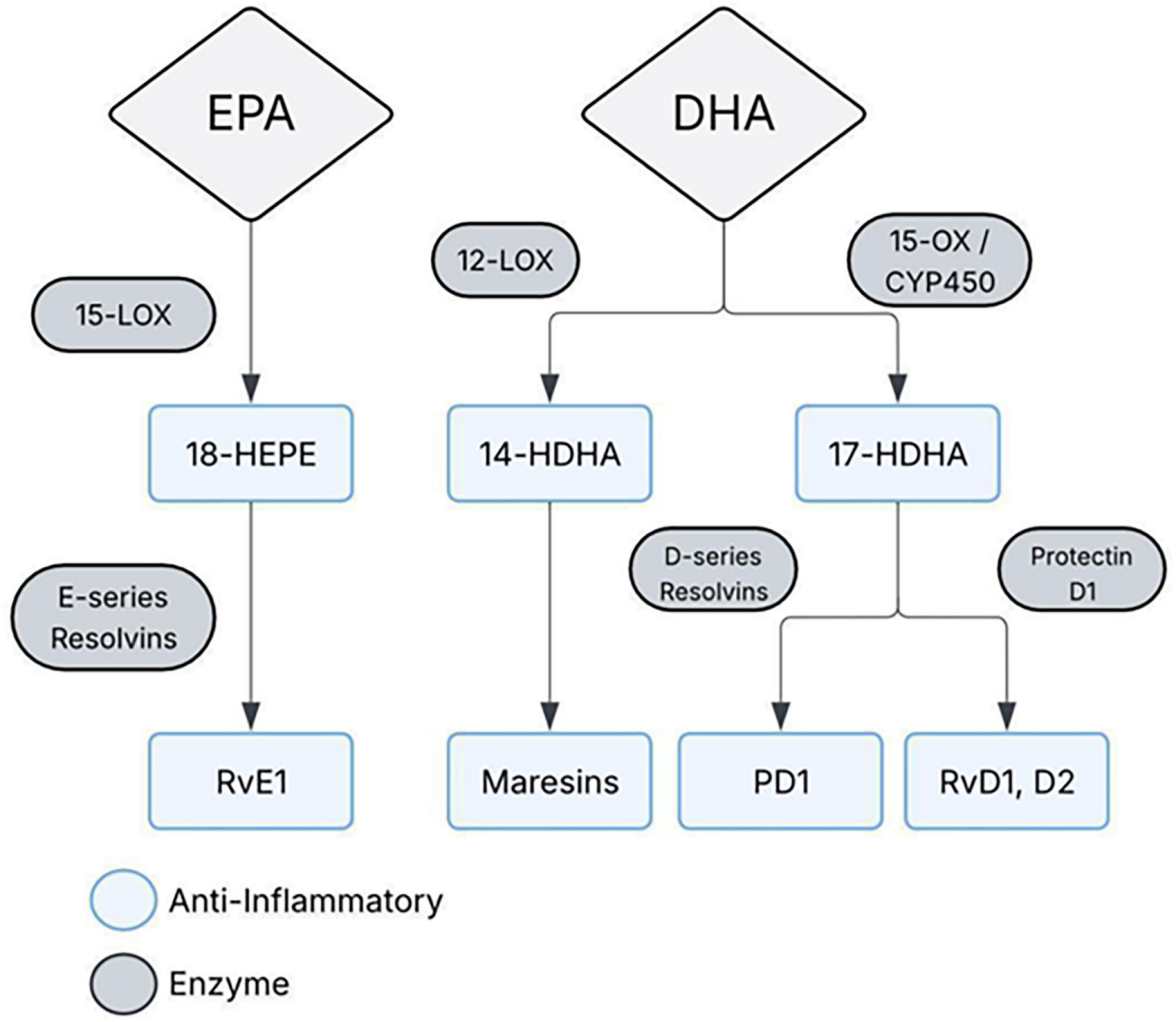
Omega-3–derived specialized pro-resolving mediators. Eicosapentaenoic acid (EPA) and docosahexaenoic acid (DHA) are converted through LOX- and CYP450-associated pathways into bioactive intermediates including 18-HEPE, 14-HDHA, and 17-HDHA. These intermediates give rise to specialized pro-resolving mediators, including E-series resolvins (RvE1), D-series resolvins (RvD1, RvD2), protectin D1 (PD1), and maresins. These lipid mediators promote resolution of inflammation and exert anti-inflammatory, tissue-protective effects.

Dietary PUFA substrate availability influences the relative production of pro-inflammatory eicosanoids versus specialized pro-resolving mediators, and diabetes-associated shifts in omega-6 and omega-3 pools may blunt pro-resolving output (25). Omega-3 enrichment restores a pro-resolving tone and attenuates injury by reducing leukocyte infiltration, oxidative stress, and endothelial dysfunction. Diabetes causes oxidative stress in the retina, which lowers brain-derived neurotrophic factor (BDNF) and harms retinal neurons. Omega-3 intake (EPA) raises its metabolite 18-HEPE (Figure 3), which has been shown in human models to increase BDNF in mouse DR and human Müller cells, protecting the retina from oxidative damage (63). Furthermore, population data link healthier PUFA patterns to lower retinopathy risk, supporting nutrition that increases marine omega-3 intake and improves overall fat quality/patterns (34).

## Clinical interventions

Early, patient-tailored glycemic control reduces microvascular complications and may help establish a favorable metabolic memory effect, likely through persistent mitochondrial oxidative stress and epigenetic marking (15). Blood pressure should be tightly controlled for all patients, with consideration of Renin-Angiotensin-Aldosterone System modulation to improve endothelial function and reduce leukocyte adhesion independent of hemodynamic effects, while aligning treatment targets and ophthalmic surveillance with current American Diabetes Association (ADA) and American Academy of Ophthalmology (AAO) Preferred Practice Patterns (PPP) guidelines (64, 65). Fenofibrate inhibits CYP2C epoxygenases and is a PPARα agonist systemically. In ocular angiogenesis models, its anti-angiogenic effect is PPARα-independent and is reversed by 19,20-epoxydocosapentaenoic acid; fenofibrate thereby reduces pro-angiogenic ω-3 epoxides (14). Consistent with this mechanism, fenofibrate suppresses retinal and choroidal neovascularization, including in PPARα^–/–^ mice, and its anti-angiogenic effect is reversed by 19,20-epoxydocosapentaenoic acid rescue (14). Fenofibrate has shown a consistent effect on slowing DR progression in large clinical trials. In the FIELD study, treatment reduced the need for first laser intervention, and in ACCORD Eye, adding fenofibrate to statin therapy lowered the rate of 4-years progression. By contrast, intensive blood-pressure control did not translate into improved ocular outcomes (66, 67). Data on statins alone are less consistent and generally do not justify retinopathy-specific prescribing in the absence of cardiovascular indications (67); that said, experimental studies offer a rationale for potential retinal benefit: simvastatin has been shown to blunt diabetes- or high-glucose–induced upregulation of VEGF and ICAM-1 and to preserve blood–retinal barrier integrity through inhibition of NADPH oxidase activity (including NOX2) and downstream STAT3 signaling (68).

Mechanistically, lipid-sensing transcriptional control (including PPAR pathways described above) likely influences inflammatory tone and barrier stability, reinforcing the rationale for systemic therapies that modulate lipid mediator biology (36). Prophylactic anti-VEGF can reduce anatomic risk in severe NPDR. PANORAMA and Diabetic Retinopathy Clinical Research (DRCR) Protocol W showed lower rates of progression to vision-threatening complications and more frequent ≥ 2-step DRSS improvements, while 2- to 4-years visual acuity advantages were not observed, which places shared decision-making at the center in eyes without DME (2, 69). Because Hcy reflects modifiable one-carbon metabolism, which is influenced by folate, vitamin B6, and vitamin B12 status (70, 71), it is prudent to screen for and correct nutritional deficiencies and manage clinically significant HHcy according to standard guidelines. While direct evidence from randomized trials showing a reduction in DR progression is limited, addressing elevated Hcy represents a rational upstream strategy to mitigate vascular and inflammatory risk in at-risk patients.

### Nutrition, exercise, and microbiome-targeted strategies

A patient’s nutrition plays a key role in addressing the metabolic stress that contributes to early DR. Dietary patterns that increase intake of marine omega-3 and reduce the omega-6 to omega-3 ratio favor a shift toward pro-resolving lipid mediators and have been associated with reduced DR risk in population studies (34, 71). In experimental models, omega-3 supplementation increases SPMs, improves neuronal function, and supports resolution pathways rather than nonspecific inflammatory suppression (72). Regular aerobic and resistance exercise further improves glycemic stability and endothelial nitric oxide signaling, which helps lower the inflammatory burden that contributes to BRB dysfunction (73–75).

**FIGURE 4:**
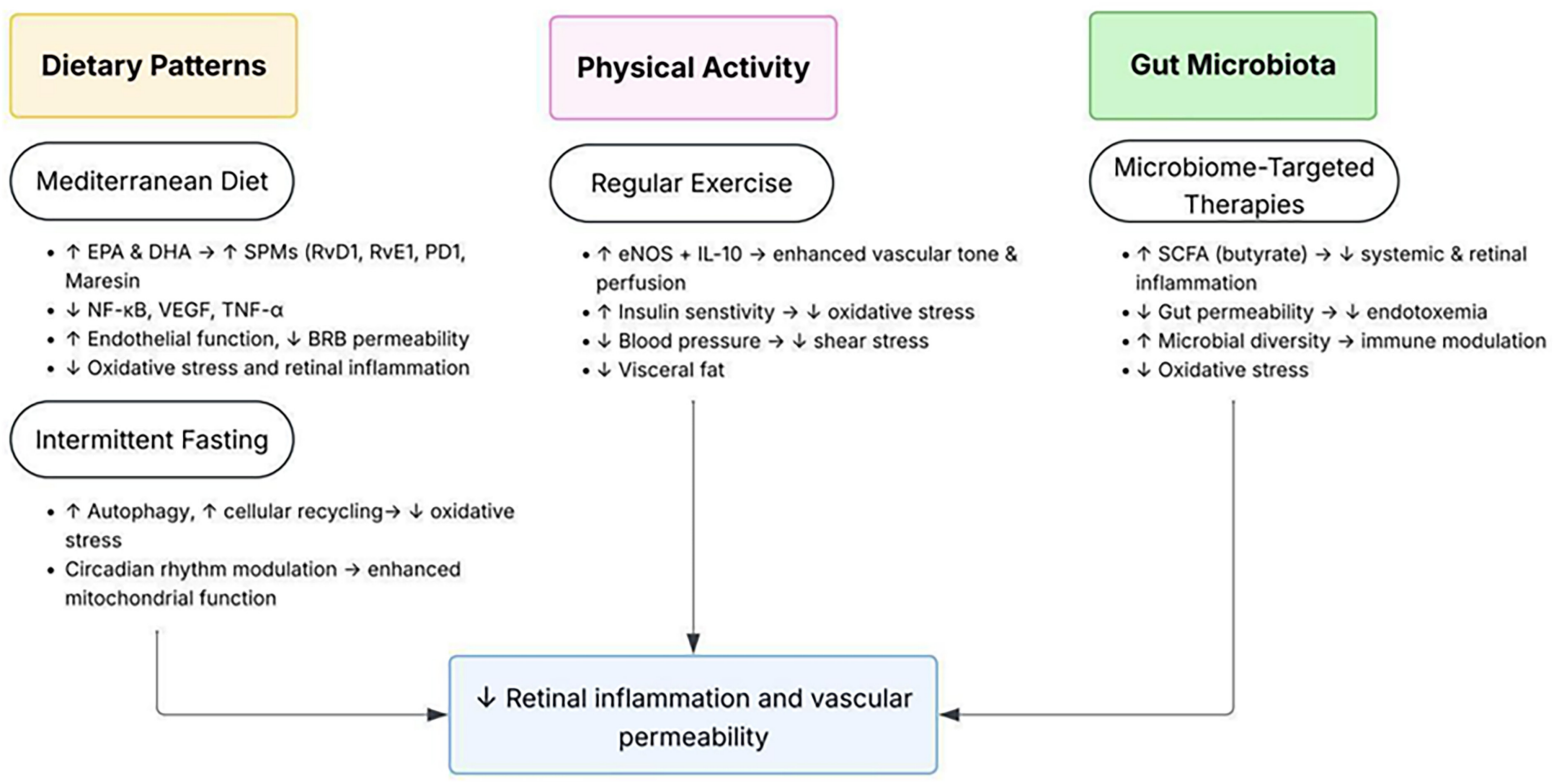
Convergent lifestyle and metabolic interventions targeting upstream mechanisms in early DR. Schematic overview illustrating how dietary patterns, physical activity, and gut microbiota modulation converge on shared metabolic and inflammatory pathways relevant to early DR. A Mediterranean dietary pattern and intermittent fasting may increase omega-3-derived specialized pro-resolving mediators, suppress NF-κB-driven inflammation, reduce oxidative stress, and help preserve blood-retinal barrier integrity. Regular physical activity improves endothelial nitric oxide signaling, insulin sensitivity, and overall vascular function while reducing visceral adiposity and systemic inflammation. Microbiome-targeted strategies enhance short-chain fatty acid production, strengthen gut barrier function, limit endotoxemia, and modulate immune responses. Together, these systemic interventions may reduce retinal inflammation and vascular permeability by addressing upstream drivers of early diabetic retinal microvascular dysfunction.

Importantly, nutrition also intersects with one-carbon metabolism because folate and B-vitamin inadequacy can contribute to elevated Hcy, which may amplify oxidative stress and inflammatory signaling relevant to BRB dysfunction (70, 76). Correcting documented deficiencies is reasonable, but DR-specific randomized outcome data for Hcy-lowering remain limited. Adherence to Mediterranean dietary patterns enriched in marine omega-3 fatty acids has been associated with reduced incidence of sight-threatening DR in prospective analyses. Intermittent fasting and caloric modulation improve insulin sensitivity and reduce systemic inflammatory tone, which may indirectly modulate retinal lipid mediator balance. Regular aerobic exercise enhances endothelial nitric oxide bioavailability and reduces oxidative stress, while emerging evidence links gut microbiome–derived short-chain fatty acids to modulation of systemic inflammation relevant to diabetic microvascular disease (77–79). These integrated mechanisms and intervention strategies are summarized in Figure 4.

#### Emerging therapies: neuroprotection and lipid-pathway modulation

Mechanistic studies support targeting lipid mediator pathways upstream. In diabetic mouse models, 12/15-LOX contributes to endoplasmic reticulum stress and inflammatory signaling via NADPH oxidase and VEGFR2; both genetic deletion and pharmacologic inhibition dampen these responses and lessen vascular injury, supporting continued development of selective LOX or GPR31 antagonists (6–9, 49). In parallel, sEH inhibitors help preserve EET levels, decrease vascular leakage, and prevent retinopathy in preclinical models, with clinical translation still in progress (56, 59). Additional strategies, such as synthetic or stabilized specialized pro-resolving mediators, neuroprotective anti-inflammatory agents, and dietary or microbiome-based interventions that shift mediator balance toward resolution, may offer complementary options for patients who continue to exhibit low-grade inflammation despite adequate systemic control (66). These programs are best viewed as adjuncts to glycemia, blood pressure, and lipid management.

## Discussion

Key uncertainties remain around the specific cell-type and timing of LOX and COX outputs in early human DR, supporting the need for direct mediator measurements and single-cell receptor mapping in human tissues. Although sEH inhibition shows robust barrier protection in mice, questions about dosing, delivery route, and potential vascular off-target effects emphasize the need for early-phase trials with ocular pharmacodynamic endpoints (56, 59). Evidence for dietary omega-3s appears stronger with food-based patterns than with some supplements, which suggests that dose, bioavailability, and indices such as the erythrocyte omega-3 level should be incorporated into future trials (12, 34). While Hcy lowering is biologically plausible, DR-specific randomized data are still limited, highlighting the need for interventional trials that pair vitamin repletion with mechanistic ocular outcomes (80). Taken together, available evidence supports a precision-prevention framework that emphasizes tight glycemic and blood pressure control, selective use of fenofibrate in appropriate type 2 diabetes phenotypes, and trial-based targeting of pathways such as 12/15-LOX or sEH in patients with high-risk molecular signatures. Prophylactic anti-VEGF therapy may be reasonable for eyes showing rapid anatomic progression or at high short-term risk due to nonadherence (2, 66, 67, 69).

Early DR reflects convergent metabolic, inflammatory, and neurovascular insults that couple systemic risk to retinal outcomes. Mechanistic data link hyperglycemia-driven oxidative stress and epigenetic memory to lipid pathway activation, including LOX and COX products that prime endothelial dysfunction and leukostasis, while elevated Hcy and dysregulated PUFA biology amplify this state (6–8, 15, 76). The most consistent clinical signals favor early individualized glycemia, steady blood pressure with RAAS consideration, and dyslipidemia management that can include fenofibrate for selected type 2 diabetes patients, alongside omega-3-forward nutrition and exercise that improve endothelial function and mediator balance (4, 34, 66, 67). Preventive anti-VEGF is appropriate for some severe NPDR eyes after shared decision-making, while experimental targeting of 12/15-LOX, GPR31, or sEH should proceed in trials with mechanistic ocular endpoints. Integrating these systemic strategies with vigilant ocular monitoring and timely local therapy offers the best chance to preserve retinal integrity and vision.

## Practice points

1. Early DR reflects imbalance between COX-, LOX-, and CYP-derived eicosanoids and pro-resolving lipid mediators.
2. Omega-3 enrichment may promote specialized pro-resolving mediator production and restore inflammatory balance.
3. Fenofibrate slows DR progression and may influence retinal lipid signaling beyond lipid lowering.
4. Homocysteine may amplify COX/LOX-mediated inflammatory signaling in high-risk phenotypes.

Emerging therapies targeting 12/15-LOX, prostanoid receptors, or soluble epoxide hydrolase remain under investigation.
